# Circulating Adiponectin and Omentin Across Cardiometabolic Phenotypes: Links to Atherogenic Indices in Prediabetes and New-Onset Type 2 Diabetes

**DOI:** 10.3390/ijms27062558

**Published:** 2026-03-11

**Authors:** Daniela Denisa Mitroi Sakizlian, Daniela Ciobanu, Lidia Boldeanu, Mohamed-Zakaria Assani, Isabela Siloși, Vlad Pădureanu, Daniel Cosmin Caragea, Venera Cristina Dinescu, Alina Elena Ciobanu Plasiciuc

**Affiliations:** 1 Doctoral School, University of Medicine and Pharmacy of Craiova, 200349 Craiova, Romania; mitroidenisa96@gmail.com (D.D.M.S.); mohamed.assani@umfcv.ro (M.-Z.A.); 2 Department of Internal Medicine, University of Medicine and Pharmacy of Craiova, 200349 Craiova, Romania; elada192@yahoo.com (D.C.); vlad.padureanu@umfcv.ro (V.P.); 3 Department of Microbiology, University of Medicine and Pharmacy of Craiova, 200349 Craiova, Romania; 4 Department of Immunology, University of Medicine and Pharmacy of Craiova, 200349 Craiova, Romania; isabela.silosi@umfcv.ro; 5 Department of Nephrology, Faculty of Medicine, University of Medicine and Pharmacy of Craiova, 200349 Craiova, Romania; daniel.caragea@umfcv.ro; 6 Department of Health Promotion and Occupational Medicine, University of Medicine and Pharmacy of Craiova, 200349 Craiova, Romania; 7 Department of Health and Motricity, Faculty of Medical and Behavioral Sciences, “Constantin Brâncuși” University of Târgu-Jiu, 210185 Târgu-Jiu, Romania; elena.ciobanu210@gmail.com

**Keywords:** adiponectin, omentin, prediabetes, type 2 diabetes mellitus, cardiometabolic phenotypes, metabolically unhealthy normal weight (MUHNW), metabolically unhealthy obese (MUHO), atherogenic index of plasma (AIP), Framingham Risk Score (FRS) 10y general CVD

## Abstract

Adiponectin and omentin are adipose tissue-derived adipokines implicated in insulin sensitivity and cardiometabolic regulation. Their behavior across different stages of dysglycemia, as well as in relation to visceral adiposity and cardiometabolic phenotypes, remains incompletely understood. In this cross-sectional study, circulating adiponectin and omentin levels were evaluated in individuals with prediabetes (PreDM, *n* = 100) and newly diagnosed type 2 diabetes mellitus (T2DM, *n* = 128). Associations with insulin resistance-related indices, including the triglyceride–glucose (TyG) index and TyG-derived composites, the visceral adiposity index (VAI), cardiometabolic phenotypes, and cardiovascular risk categories, were assessed using correlation and multivariable regression analyses. Discriminatory performance for metabolically unhealthy obesity was evaluated using receiver operating characteristic (ROC) curve analysis. Both adiponectin and omentin levels were lower in T2DM compared with PreDM (22.05 vs. 30.30 and 25.72 vs. 38.84, *p* < 0.0001 for both). In PreDMs, omentin showed a significant inverse correlation with the TyG index (weak correlation, *ρ* = −0.197, *p* = 0.050), whereas adiponectin demonstrated only weak trends. In multivariable models, VAI and male sex were independent predictors of circulating omentin levels, whereas fasting insulin was not. In contrast, adiponectin did not retain independent associations with metabolic or visceral adiposity indices. In T2DM, adipokine–metabolic associations were largely absent. Neither adipokine differed substantially across cardiometabolic phenotypes or cardiovascular risk categories. ROC analyses revealed modest overall discriminatory performance for metabolically obese phenotypes, with poor discrimination after stratification by glycemic status (area under the ROC curve (AUC) of 0.704 for adiponectin and 0.710 for omentin, and AUC of 0.431 for adiponectin and 0.461 for omentin, respectively). Circulating adipokines appear to exhibit stage-dependent relationships with metabolic dysfunction, being more informative in PreDM than in established T2DM. Omentin may reflect visceral adiposity-related metabolic alterations in early dysglycemia, whereas adiponectin shows limited independent associations. Overall, these findings suggest that adipokines have limited diagnostic or cardiovascular risk-stratification utility when considered in isolation and may be better interpreted within multimarker cardiometabolic assessment frameworks.

## 1. Introduction

Cardiometabolic disorders represent a major global health burden, driven by obesity, dyslipidemia, and impaired glucose metabolism [1]. Prediabetes (PreDM) and newly diagnosed type 2 diabetes mellitus (T2DM) represent early but critical stages in which cardiometabolic abnormalities begin to cluster, substantially increasing long-term cardiovascular risk. Importantly, cardiometabolic risk is not uniformly determined by body mass index (BMI), as individuals with similar BMI values may exhibit markedly different metabolic profiles [1,2].

To better capture this heterogeneity, the concept of cardiometabolic phenotypes has emerged beyond simple anthropometric measures. Individuals classified as metabolically unhealthy normal weight (MUHNW) exhibit insulin resistance (IR), dyslipidemia, and increased cardiovascular risk despite normal BMI, whereas metabolically unhealthy obese (MUHO) individuals combine excess adiposity with overt metabolic dysfunction. This phenotype-based framework underscores the limitations of BMI-based risk stratification and highlights the dissociation between adiposity and metabolic health [1,3]. Recently, Soheilifard and colleagues proposed a refined classification of cardiometabolic phenotypes that integrates metabolic health and adiposity, demonstrating strong associations with the atherogenic index of plasma (AIP) and dietary patterns in a large population-based cohort. This framework provides a robust approach for evaluating atherogenic risk in both normal-weight and obese individuals with metabolic dysfunction [4,5].

Observational evidence indicates that MUHNW is associated with elevated cardiovascular events and mortality, suggesting that “normal weight” is not necessarily metabolically benign [6,7]. In addition, comparative data suggest that MUHNW may exhibit cardiometabolic vulnerability comparable to, or even greater than, MUHO [8].

Adipose tissue is a dynamic endocrine organ that secretes adipokines regulating glucose homeostasis, lipid metabolism, inflammation, and vascular function [9,10]. Adiponectin is widely recognized for its insulin-sensitizing, anti-inflammatory, and anti-atherogenic properties [10,11,12]. Circulating adiponectin levels are consistently lower in individuals with obesity, T2DM, and cardiovascular disease (CVD), indicating its role as both a marker and a possible mediator of cardiometabolic risk [12,13,14]. Studies indicate adiponectin influences lipid oxidation, endothelial health, and glucose balance, acting as a protective marker in cardiometabolic health diseases. The adipocytokines leptin and adiponectin have been variously associated with diabetic microvascular complications [15,16,17,18,19,20,21].

Omentin-1 (intelectin-1), predominantly expressed in visceral adipose tissue, has been linked to improved insulin sensitivity and anti-inflammatory effects, with lower circulating levels reported in obesity and IR states [22,23,24,25]. Compared with adiponectin, omentin-1 may reflect more closely visceral adipose tissue dysfunction and early metabolic disturbances, particularly in the context of dysglycemia. Research has shown lower omentin-1 levels in T2DM patients with metabolic-associated fatty liver disease (MAFLD), along with higher fasting plasma glucose and decreased high-density lipoprotein cholesterol (HDL-C) levels [26]. A study found individuals without metabolic syndrome had higher omentin-1 levels than those with it, suggesting omentin-1 may protect against metabolic issues [27]. Evidence suggests an inverse correlation between omentin-1 levels and insulin resistance markers, with elevated BMI associated with decreased omentin-1 concentrations. According to prior studies, plasma omentin levels demonstrate an inverse relationship with BMI, fat mass, and fasting plasma insulin, while maintaining a positive correlation with insulin sensitivity, adiponectin, HDL-C, and endothelial function [28,29]. However, an increasing body of literature has shown paradoxical relationships between serum omentin and diabetes, with some studies suggesting a significant association [30,31,32], while others do not [33,34,35,36].

The Visceral Adiposity Index (VAI) is a sex-specific composite marker integrating anthropometric and lipid parameters to better estimate visceral adipose tissue dysfunction than BMI alone. VAI has been associated with insulin resistance, components of metabolic syndrome, incident T2DM, and cardiovascular risk across diverse populations, supporting its role as a practical surrogate marker of adipose dysfunction in early dysglycemia [37,38,39,40,41].

In individuals with PreDMs and newly diagnosed T2DM, atherogenic dyslipidemia, characterized by elevated triglycerides (TGs) and reduced HDL-C, is common and contributes to cardiovascular risk. Composite lipid-derived indices, such as the AIP, TG/HDL-C ratio, and lipid accumulation product (LAP), integrate lipid and anthropometric parameters and have shown superior predictive value for cardiovascular events compared with traditional lipid markers, particularly in PreDM and T2DM [42,43]. These indices may be especially informative for cardiometabolic phenotypes such as MUHNW, where traditional lipid measures and BMI-based assessments may underestimate the atherogenic burden.

Despite increasing interest in cardiometabolic phenotypes and visceral adiposity, limited data exist regarding stage-dependent differences in adiponectin and omentin across MUHNW and MUHO phenotypes in early dysglycemia, and how these adipokines relate to composite atherogenic indices and visceral adiposity measures across these phenotypes. Therefore, the aim of this study was to compare circulating adiponectin and omentin concentrations between MUHNW and MUHO individuals with PreDM and newly diagnosed T2DM, and to evaluate their associations with atherogenic lipid indices and visceral adiposity, in order to refine cardiometabolic phenotyping beyond BMI-based classification.

## 2. Results

### 2.1. Baseline Characteristics of Participants with Prediabetes and Newly Diagnosed Type 2 Diabetes

Table 1 presents an overview of the anthropometric and metabolic features of the study participants. Participants with PreDM and those with newly diagnosed T2DM were comparable in age, with no statistically significant difference between groups (57.83 ± 10.89 vs. 55.41 ± 11.53 years, *p* = 0.185). Similarly, place of residence (rural/urban), sex distribution, and educational status did not differ significantly between groups, indicating a broadly comparable demographic background.

In contrast, lifestyle-related factors differed markedly. Alcohol consumption and smoking were significantly more prevalent in the PreDMs group, whereas a higher proportion of individuals with T2DM were non-drinkers and non-smokers (*p* = 0.003 and *p* = 0.002, respectively). These differences may reflect behavioral changes following diabetes diagnosis or increased health awareness in patients with established disease.

The prevalence of hepatic steatosis, dyslipidemia, and hypertension was high in both groups, with no significant between-group differences, underscoring the substantial cardiometabolic burden already present at the PreDMs stage. T2DM exhibited markedly higher SBP compared with the PreDM group.

Anthropometric parameters revealed pronounced differences between groups. Anthropometric measures indicated higher body weight and BMI in T2DM (both *p* ≤ 0.002), as well as higher hip circumference and a greater waist-to-hip ratio (WHR). This finding suggests that patterns of fat distribution, rather than absolute adiposity, may differ between metabolic states.

As expected, glycemic parameters were markedly worse in the T2DM group, with significantly higher glycosylated hemoglobin A1c (HbA1c) and fasting plasma glucose (FPG) levels (*p* < 0.0001 for both), confirming clear metabolic separation between PreDM and T2DM.

In contrast, lipid parameters showed higher TC, LDL-C, and non-HDL-C in PreDM (all *p* < 0.05), whereas TG and HDL-C were similar between groups.

Profound differences emerged when evaluating insulin resistance (IR) and sensitivity indices. Individuals with T2DM exhibited markedly higher fasting insulin levels and Homeostatic Model Assessment of Insulin Resistance (HOMA-IR), alongside substantially lower Quantitative Insulin Sensitivity Check Index (QUICKI) values (*p* < 0.0001 for all), reflecting severe insulin resistance in the diabetic state.

Among lipid-derived indices, triglyceride–glucose (TyG) and TyG-BMI were significantly higher in T2DM, emphasizing the combined effect of dysglycemia and adiposity on insulin resistance (*p* = 0.041 and *p* < 0.0001), whereas TG/HDL-C ratio, AIP, TyG-WC, and TyG-WHtR did not differ significantly, suggesting heterogeneous performance across metabolic stages. VAI values were significantly higher in the PreDM group compared with newly diagnosed T2DM (median 3.01 vs. 2.18; Mann–Whitney U test, *p* = 0.004).

Cardiovascular risk was substantially higher in individuals with T2DM, as reflected by both the 10-year general CVD risk from Framingham Risk Score (FRS) and the World Health Organization (WHO) CVD risk scores (*p* < 0.0001 for both). Importantly, participants with PreDM already exhibited moderate predicted CVD risk, highlighting that increased cardiovascular vulnerability precedes the onset of overt diabetes.

The distribution of cardiometabolic phenotypes differed significantly between groups (*p* = 0.023). While metabolically healthy normal weight (MHNW) and MUHNW phenotypes were prevalent in PreDM, the T2DM group was dominated by metabolically healthy obese (MHO) and MUHO phenotypes, illustrating the shift toward obesity-associated metabolic dysfunction with disease progression.

**Figure 1:**
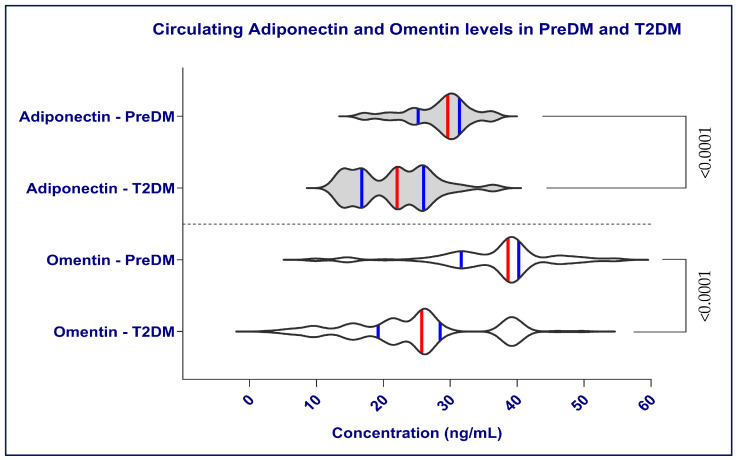
Circulating adiponectin and omentin levels in prediabetes (PreDM) and type 2 diabetes mellitus (T2DM). The violin plots display the distribution of adiponectin and omentin in both groups. Vertical red lines represent medians, while blue vertical lines indicate the interquartile range (IQR). Values are shown in ng/mL. The violin plots show higher serum adiponectin and omentin levels in PreDM subjects, with a statistically significant difference (*p* < 0.0001) according to the Mann–Whitney U test.

Both adiponectin and omentin levels were significantly lower in individuals with T2DM compared with those with PreDM (*p* < 0.0001 for both) (Figure 1). These findings support the concept that progression from PreDM to diabetes is accompanied by a decline in protective adipokines, potentially contributing to worsening IR and cardiovascular risk.

### 2.2. Comparing the Adipokines and Metabolic Parameters Levels in the PreDM and T2DM Groups

#### 2.2.1. Sex-Related Differences in Adipokines and Metabolic Risk Profiles in PreDM and T2DM

Table 2 presents a comparison of adipokines and metabolic parameters by gender within the PreDM and T2DM groups.

##### Prediabetes Group

Sex-specific analyses revealed significant differences in adipokines and metabolic indices, primarily in the PreDM group. In the PreDM group, both adiponectin (31.84 [24.75–38.72] vs. 28.89 [26.95–30.96] ng/dL, *p* = 0.019), and omentin (41.42 [38.54–43.50] vs. 36.29 [30.36–41.74] ng/mL, *p* = 0.013), were significantly higher in males compared with females, indicating a comparable adiponectin profile across sexes at this early stage of dysglycemia.

QUICKI was also higher in males (*p* = 0.026), indicating a broadly similar degree of IR between males and females in the PreDM group. A marked sex difference was observed for cardiovascular risk estimates (FRS and WHO CVD risk), which were consistently higher in males (16.63 [9.53–26.38] vs. 9.24 [6.15–13.83] %, *p* <0.0001; 12.50 [8.25–17.00] vs. 10.00 [9.40–14.75] %, *p* = 0.016).

Females showed higher composite TyG-derived indices (TyG-BMI and TyG-WHtR), suggesting a potential sex-specific association between IR and central adiposity when WHtR is considered.

##### Type 2 Diabetes Mellitus Group

In patients with newly diagnosed T2DM, no significant sex differences were observed in adiponectin or omentin levels, indicating that sex-related differences in adipokine secretion appear attenuated once overt diabetes is established.

Similarly, fasting insulin, HOMA-IR, and QUICKI did not differ significantly between males and females, reflecting comparable degrees of IR in both sexes in the diabetic state.

Despite these similarities in metabolic control, cardiovascular risk differed markedly by sex. Males displayed a substantially higher FRS compared with women (34.68 [22.94–50.09] vs. 21.91 [12.08–31.59] %, *p* < 0.0001), underscoring a pronounced male disadvantage in predicted 10-year cardiovascular risk. In addition, the WHO CVD risk was significantly higher in males than in females (20.00 [17.00–22.00] vs. 18.00 [15.00–22.00] %, *p* = 0.018).

Traditional lipid-derived indices, including TG/HDL-C, AIP, TyG, TyG-BMI, and TyG-WC, did not show statistically significant sex differences. Notably, however, TyG-WHtR remained significantly higher in females (6.05 ± 0.80 vs. 5.69 ± 0.74, *p* = 0.009), mirroring the pattern observed in the PreDM group and suggesting a consistent sex-specific relationship between IR and body fat distribution.

In both glycemic groups, females exhibited significantly higher VAI values compared with males. In the PreDM group, median VAI was 3.87 [2.94–5.32] in females vs. 3.09 [2.13–4.26] in males (*p* = 0.007). Similarly, in newly diagnosed T2DM, females demonstrated higher VAI values than males (2.78 [2.13–3.60] vs. 1.79 [1.25–2.45], *p* < 0.0001), indicating a consistent sex-specific pattern of visceral adiposity dysfunction.

#### 2.2.2. Adipokines and Metabolic Parameters Across Cardiometabolic Phenotypes in the PreDM Group

Table 3 summarizes the distribution of circulating adipokines and insulin resistance-related parameters (row factor) across cardiometabolic phenotypes (column factor), within the PreDM group, namely metabolically healthy normal-weight (MHNW), MUHNW, and MUHO, using a two-way ANOVA (Kruskal–Wallis) test.

Circulating adiponectin levels did not differ significantly across cardiometabolic phenotypes in individuals with PreDM (column factor: F(1,55) = 0.183, *p* = 0.671). Similarly, omentin concentrations were comparable across phenotypes (column factor: F(1,55) = 0.044, *p* = 0.835). This finding indicates that, within PreDMs, differences in metabolic health or adiposity are not yet associated with marked alterations in circulating omentin levels. Adipokines may reflect global metabolic processes rather than discrete phenotypic classifications.

In contrast, markers of insulin resistance showed significant differences. Fasting insulin levels were significantly higher in the MUHO phenotype compared with MHNW and MUHNW (column factor: F(1,55) = 3.500, *p* = 0.046), while QUICKI was significantly lower in MUHO (column factor: F(1,55) = 4.833, *p* = 0.032), indicating reduced insulin sensitivity. Although HOMA-IR increased numerically across phenotypes, this difference did not reach statistical significance.

As summarized in Table 3, the MUHO phenotype exhibits higher insulin resistance, but adipokine concentrations did not differ significantly across cardiometabolic phenotypes. These results suggest that IR more accurately reflects the metabolic heterogeneity of phenotypes, whereas adipokines may reflect the overall stage of dysglycemia rather than the specific metabolic phenotype.

#### 2.2.3. Adipokines and Metabolic Parameters Across Cardiometabolic Phenotypes in the T2DM Group

Table 4 presents the distribution of circulating adipokines and insulin resistance-related parameters (row factor) across cardiometabolic phenotypes (column factor) in patients with newly diagnosed T2DM, including MHO and MUHO.

No significant differences were observed in adiponectin levels between MHO and MUHO individuals (median [IQR]: 22.15 [16.62–26.11] vs. 22.05 [16.77–25.96] ng/dL; column factor: F(1,55) = 0.183, *p* = 0.671). This finding suggests that, in the context of overt diabetes, circulating adiponectin concentrations are similarly reduced regardless of metabolic health status among obese individuals. Likewise, omentin levels were comparable between the two phenotypes (column factor: F(1,55) = 0.044, *p* = 0.835), indicating that phenotype-specific differences in metabolic health do not translate into differential omentin secretion once T2DM is established.

In contrast to adipokines, fasting insulin levels differed significantly between phenotypes, with higher insulin concentrations in MUHO than in MHO individuals (column factor: F(1,55) = 3.500, *p* = 0.046). This finding reflects a more pronounced hyperinsulinemic state in MUHO patients.

Although HOMA-IR values were numerically higher in the MUHO group, this difference did not reach statistical significance (column factor: F(1,55) = 2.171, *p* = 0.146), suggesting substantial overlap in IR severity between obese phenotypes. Notably, insulin sensitivity, as assessed by QUICKI, was significantly lower in MUHO individuals compared with MHO individuals (column factor: F(1,55) = 4.833, *p* = 0.032), highlighting a phenotype-specific impairment in insulin sensitivity that is not fully captured by HOMA-IR alone.

Row factor analysis revealed no significant within-phenotype variability in adiponectin, omentin, insulin, HOMA-IR, or QUICKI (all *p* > 0.05), indicating consistent distributions across cardiometabolic phenotypes.

### 2.3. Associations Between Adipokines and Metabolic and Atherogenic Indices

Correlation analyses using Spearman’s rank test were performed to evaluate the associations between circulating adipokines (adiponectin and omentin) and lipid-derived and insulin resistance-related indices in participants with PreDM and newly diagnosed T2DM.

#### 2.3.1. Prediabetes Group

**Figure 2:**
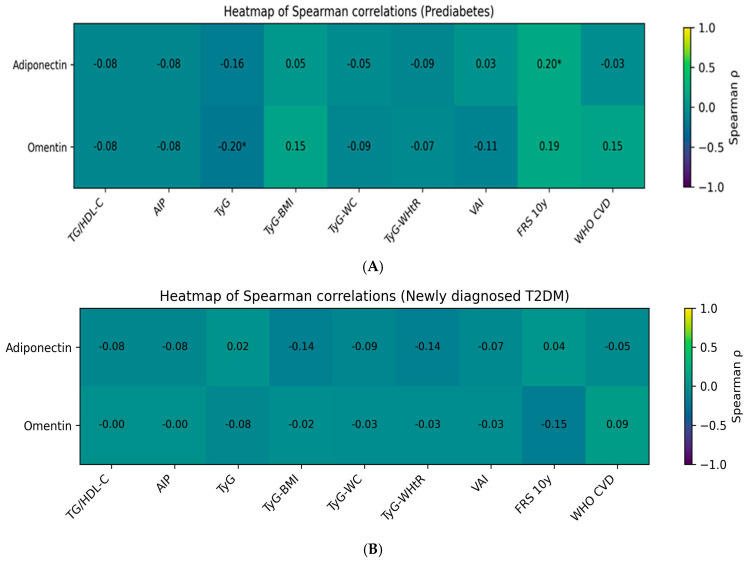
Heatmap of Spearman correlations between circulating adipokines and metabolic and atherogenic indices, in (**A**) Prediabetes (PreDM) group, (**B**) Newly diagnosed type 2 diabetes mellitus (T2DM) group. Heatmaps illustrate the strength and direction of Spearman’s rank correlation coefficients (*ρ*) between circulating adipokines (adiponectin and omentin) and lipid-derived and insulin resistance-related, and composite cardiometabolic indices, including the triglyceride-to-HDL cholesterol ratio (TG/HDL-C), the atherogenic index of plasma (AIP), the triglyceride–glucose index (TyG), and TyG-based composite indices incorporating anthropometric measures (TyG-BMI, TyG-WC, and TyG-WHtR), the visceral adiposity index (VAI), and cardiovascular risk scores (Framingham Risk Score 10-year risk [FRS 10y] and WHO CVD risk score). Color intensity reflects the magnitude of the correlation coefficient, with negative correlations shown in cooler tones and positive correlations in warmer tones. Numerical values within each cell represent the corresponding Spearman *ρ* coefficient. Statistical significance is indicated by asterisks (* *p* < 0.05). In the PreDM group (**A**), a modest positive association between adiponectin and the FRS 10y, and a significant inverse association between omentin and the TyG index were observed, indicating lower omentin concentrations in individuals with greater insulin resistance. No significant correlations were detected between adipokines and VAI. No other adipokine–index associations reached statistical significance. In the T2DM group (**B**), correlations between adipokines and atherogenic or insulin resistance, visceral adiposity, or cardiovascular risk indices were uniformly weak and did not reach statistical significance, suggesting attenuation of adipokine–metabolic relationships in the context of established diabetes. BMI, body mass index; WC, waist circumference; WHtR, waist-to-height ratio.

In individuals with PreDM (Table 5, Figure 2A), adiponectin levels were not significantly correlated with classical atherogenic lipid indices, including the TG/HDL-C ratio and the AIP (*ρ* = −0.077, *p* = 0.448 for both). No significant associations were observed between adiponectin and composite indices integrating anthropometric parameters, including TyG, TyG-BMI, TyG-WC, and TyG-WHtR (*p* > 0.05 for all). Also, adiponectin did not show statistically significant correlations with VAI (*ρ* = 0.029, *p* = 0.771) or with WHO CVD risk (*ρ* = −0.032, *p* = 0.750), except for a modest positive association with the FRS 10y cardiovascular risk score (*ρ* = 0.203, *p* = 0.042).

In contrast, omentin levels showed a significant inverse correlation with the TyG index (weak correlation, *ρ* = −0.197, *p* = 0.050), indicating that lower omentin concentrations are associated with greater IR in PreDMs. No significant correlations were identified between omentin and lipid-based atherogenic indices or anthropometry-adjusted TyG derivatives (*p* > 0.05). Also, omentin correlations with VAI and cardiovascular risk scores (FRS 10y and WHO CVD risk) were not statistically significant.

#### 2.3.2. Type 2 Diabetes Mellitus Group

In patients with newly diagnosed T2DM (Table 6, Figure 2B), neither adiponectin nor omentin levels were significantly correlated with atherogenic lipid ratios, including TG/HDL-C and AIP (*p* > 0.35 for all). Similarly, no significant associations were observed between adipokines and TyG-based indices (TyG, TyG-BMI, TyG-WC, or TyG-WHtR).

Overall, the correlation patterns observed in PreDM were no longer evident in the T2DM group, suggesting attenuation of adipokine–metabolic relationships with progression to overt diabetes.

Taken together, these findings indicate that omentin is more closely linked to insulin resistance in the PreDM stage, as reflected by its significant inverse correlation with the TyG index, whereas adiponectin shows only weak or non-significant associations with atherogenic and IR indices in this cohort. In contrast, no meaningful adipokine–atherogenic index associations were detected in newly diagnosed T2DM, suggesting that adipokine signaling may be overshadowed by advanced metabolic dysregulation once diabetes is established.

In newly diagnosed T2DM, adipokines did not show significant correlations with VAI (adiponectin and omentin; *ρ* = −0.070, *p* = 0.452, and *ρ* = −0.030, *p* = 0.747, respectively), or cardiovascular risk scores (FRS 10-year and WHO CVD risk).

### 2.4. Multivariable Regression Analysis

To assess whether the association between omentin and IR, as reflected by the TyG index, is independent of potential confounders, a multivariable linear regression (MLR) analysis was performed in the PreDM group, where a significant univariate association had been identified.

Circulating omentin levels were included as the dependent variable, while the TyG index was entered as the main independent predictor. The model was adjusted for age, sex, BMI, and VAI. All variables were entered simultaneously using the enter method.

#### Multivariable Regression Analysis with Omentin as the Dependent Variable

In Model A (TyG + age + sex + BMI) (Table 7), the inverse association between omentin and the TyG index observed in univariate analysis was attenuated and did not remain statistically significant after adjustment (*β* = −2.48, 95% CI: −6.38 to 1.41, *p* = 0.208). This finding suggests that the univariate association observed between omentin and IR in PreDM is partially influenced by covariates, particularly sex-related differences. In contrast, BMI emerged as an independent positive predictor of circulating omentin levels (*β* = 0.58, 95% CI: 0.17–0.99, *p* = 0.006). Age and sex were not significantly associated with omentin concentrations in this model (*p* > 0.05).

The overall model explained a modest proportion of variance in omentin levels (adjusted R^2^ = 0.105), indicating that additional factors beyond IR and basic anthropometric measures may contribute to circulating omentin variability in PreDM.

To specifically address visceral adiposity, BMI was replaced by the VAI in Model B (TyG + age + sex + VAI). In this model, both the TyG index (*β* = −7.64, *p* = 0.002) and VAI (*β* = 0.93, *p* = 0.002) emerged as independent predictors of circulating omentin levels, whereas age and sex were not significant contributors. The inclusion of VAI modestly increased the explanatory power of the model (R^2^ = 0.122), supporting the hypothesis that visceral adiposity plays a relevant role in modulating circulating omentin concentrations in PreDM.

In Model C (Insulin + age + sex + VAI), fasting insulin was entered as the primary metabolic predictor. After adjustment, fasting insulin was independently associated with circulating omentin levels (*β* = 0.35, 95% CI: 0.06–0.64, *p* = 0.017). In contrast, VAI was not significantly associated with omentin concentrations in this model, and neither age nor sex showed independent effects (*p* > 0.05). The overall explanatory power of Model C remained modest (R^2^ = 0.086).

Taken together, these extended models suggest that in individuals withPreDM:

- The univariate association between omentin and TyG is partially influenced by adiposity-related variables.
- BMI and VAI appear to contribute to omentin variability, highlighting the potential role of adiposity in modulating circulating omentin levels.
- Fasting insulin also shows an independent association with omentin, indicating a potential link between adipokine regulation and insulin dynamics in early dysglycemia.
- Adiponectin does not show independent associations with IR or visceral adiposity markers in adjusted analyses.

These findings further support the concept that ometin levels in prediabetes may reflect complex interactions between adiposity and metabolic regulation rather than isolated insulin resistance markers.

The absence of significant correlations between circulating adipokines and insulin resistance-related indices in the T2DM group may reflect stage-dependent alterations in adipokine biology. In established diabetes, adiponectin and omentin levels are markedly reduced and exhibit limited interindividual variability, suggesting a potential floor effect that diminishes their discriminatory capacity. Moreover, chronic IR, glucotoxicity, and systemic inflammation may overshadow adipokine-mediated metabolic regulation, leading to dissociation between circulating adipokine levels and metabolic indices. Together, these findings support the concept that adipokines are more informative markers of metabolic dysregulation in early stages of dysglycemia, such as PreDM, than in overt T2DM.

### 2.5. Discriminatory Performance of Adipokines for Metabolic Phenotypes

The discriminatory ability of circulating adipokines to identify metabolically obese phenotypes was evaluated using ROC curve analysis. ROC analyses were performed in the overall study population and stratified by glycemic status (Table 8).

**Figure 3:**
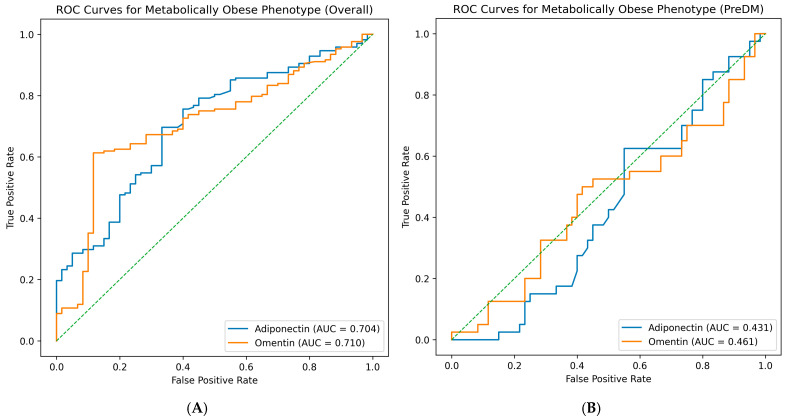
Receiver operating characteristic (ROC) curves for the discrimination of metabolically obese phenotypes using circulating adipokines, in the overall study population (**A**) and stratified by glycemic status. (**B**) ROC curves illustrate the discriminatory performance of adiponectin and omentin in identifying metabolically obese phenotypes. The area under the curve (AUC) indicates the ability of each adipokine to distinguish metabolically obese from non-obese individuals. The green dashed line represents the reference line corresponding to random classification (AUC = 0.5).

In the overall cohort (Figure 3A), both adiponectin and omentin demonstrated modest discriminatory ability. Adiponectin yielded an area under the ROC curve (AUC) of 0.704 (95% CI: 0.628–0.774), with an optimal cut-off value of 26.37 µg/mL, corresponding to a sensitivity of 70% and specificity of 67%. Similarly, omentin showed a comparable discriminatory performance with an AUC of 0.710 (95% CI: 0.636–0.781). The optimal cut-off value for omentin was 28.76 ng/mL, providing a sensitivity of 61% and specificity of 88%. These findings indicate that both adipokines exhibit only moderate ability to distinguish metabolically obese from non-obese individuals when the entire study population is considered.

When the analysis was restricted to the PreDM group (Figure 3B), the discriminatory performance of both adipokines markedly decreased. In this subgroup, adiponectin showed an AUC of 0.431 (95% CI: 0.323–0.544), with an optimal cut-off value of 29.87 µg/mL (sensitivity 63%, specificity 45%). Omentin demonstrated similarly poor discriminatory capacity, with an AUC of 0.461 (95% CI: 0.345–0.573) and an optimal cut-off value of 38.55 ng/mL (sensitivity 50%, specificity 58%). These AUC values approach random classification, indicating that adiponectin and omentin do not provide meaningful discrimination of metabolically obese phenotypes within the PreDM subgroup.

In the T2DM group, ROC analysis was not applicable due to the predominance of the metabolically obese individuals and insufficient representation of metabolically healthier profiles.

Taken together, these findings indicate that the modest discriminatory performance of adipokines observed in the overall cohort is largely driven by between-group differences and does not persist within specific glycemic strata. This suggests that adipokines reflect global metabolic burden rather than robust phenotype-specific classification, supporting their role as biological markers of metabolic dysfunction rather than diagnostic tools for cardiometabolic phenotypes.

## 3. Discussion

The present study provides an integrated evaluation of circulating adiponectin and omentin across the spectrum of dysglycemia, combining cardiometabolic phenotypes, IR-related indices, cardiovascular risk scores, and ROC-based discrimination analyses. The main finding is that adipokine–metabolic relationships appear stage-dependent, being more evident in PreDMs and largely attenuated in newly diagnosed T2DM.

### 3.1. Differential Behavior of Adipokines Across Stages of Dysglycemia

A central observation of the present study is the progressive decline in circulating adiponectin and omentin levels from PreDMs to newly diagnosed T2DM. This trajectory is consistent with population-based evidence showing a progressive decrease in adiponectin across normoglycemia, prediabetes, and diabetes, supporting its role as a marker of worsening metabolic health [44]. Mechanistically, suppressed adiponectin signaling has been linked to cardiometabolic dysfunction in diabetes, including impaired AMPK-related pathways and adverse vascular effects [45,46].

For omentin, prior systematic reviews and meta-analyses consistently report lower circulating omentin levels in T2DM and in individuals with abnormal glucose tolerance, consistent with its proposed insulin-sensitizing and anti-inflammatory actions [47,48]. In the PreDMs group, omentin exhibited a significant inverse correlation with the TyG index, a validated surrogate marker of IR, whereas no such association was observed in the T2DM group. This association was not observed for adiponectin and was absent in the T2DM group. These findings suggest that omentin may be particularly sensitive to early IR, preceding overt diabetes, whereas adiponectin alterations may reflect more global or chronic metabolic changes. More recent clinical studies also support its association with central obesity and dysglycemia, including sex-related patterns [49,50].

Importantly, our results suggest stage-dependent relevance: in PreDMs, omentin correlated inversely with the TyG index (an IR surrogate), whereas in T2DM, the adipokine–index correlations were largely absent. This aligns with the concept that, once diabetes is established, IR becomes strongly multifactorial (glucotoxicity, lipotoxicity, inflammation), potentially obscuring linear associations with single circulating adipokines [45,47].

The attenuation of adipokine–metabolic associations in newly diagnosed T2DM highlights a critical transition point. In overt diabetes, IR becomes multifactorial, driven by glucotoxicity, lipotoxicity, and chronic low-grade inflammation, potentially overshadowing the contribution of individual adipokines. This dissociation supports the concept that adipokines are more informative markers of early metabolic dysregulation than of established diabetes.

### 3.2. Adipokines, Cardiometabolic Phenotypes, and Insulin Resistance

Phenotype-based analyses showed limited differences in adipokine levels across cardiometabolic phenotypes, particularly in PreDMs, underscoring the heterogeneity of early dysglycemia, where metabolic dysfunction may precede or occur independently of phenotype-based classifications such as metabolically unhealthy obesity. This is compatible with modern views that “metabolically healthy obesity” is often transient and is strongly influenced by visceral adiposity and an inflammatory burden rather than by BMI alone [51,52]. In contrast, within the T2DM group, insulin-related parameters, but not adipokine concentrations, differed between metabolically healthy obese and metabolically unhealthy obese individuals. This observation further supports the notion that adipokine dysregulation may occur early and plateau once diabetes is established, whereas phenotype-related differences in insulin sensitivity continue to evolve.

In this context, TyG-derived indices (including those incorporating adiposity measures) may capture IR and adipose dysfunction more robustly than isolated lipid markers. Large-scale, contemporary studies support the clinical utility of TyG and TyG–adiposity composites as practical proxies for IR and broader metabolic risk [53,54]. Moreover, recent work suggests TyG relates not only to IR but also to low-grade inflammation and adipokine imbalance—supporting its biological plausibility as a “bridging” marker between metabolic and inflammatory dysregulation [55].

The multivariable regression analyses reinforce this interpretation. Our multivariable models further indicate that the univariate omentin–TyG association in PreDMs is attenuated after adjustment, while sex remains an independent determinant of omentin. This is consistent with newer evidence emphasizing sex- and fat distribution-dependent differences in omentin physiology and its links to central obesity and glucose tolerance [49,50].

### 3.3. Adipokines and Cardiovascular Risk: Limited Discriminatory Value of Global Risk Scores

An important finding of the present study is the lack of a strong association between circulating adipokine levels and the global cardiovascular risk categories defined by the FRs.

Although individuals classified in higher cardiovascular risk strata exhibited numerically lower adiponectin concentrations, these differences were modest and characterized by substantial overlap across risk categories. Omentin levels, in turn, remained largely comparable across cardiovascular risk classifications.

In line with our correlation analyses, circulating adipokines showed limited association with global cardiovascular risk scores, which are predominantly driven by age, blood pressure, lipid levels, and smoking status. Unlike metabolic indices that capture IR and adiposity-related dysfunction, composite cardiovascular risk scores aggregate multiple heterogeneous risk factors, potentially diluting the contribution of adipose tissue-derived signals. Contemporary reviews emphasize that adiponectin’s cardiovascular associations can be complex, context-dependent, and nonlinear, particularly in diabetes and cardiometabolic disease [46,56].

The weak stratification of adipokines across cardiovascular risk categories may also reflect a temporal dissociation between metabolic and cardiovascular processes. Adiponectin and omentin primarily regulate insulin sensitivity, endothelial function, and anti-inflammatory pathways, which may exert their protective effects early in the course of metabolic disease, before the accumulation of traditional cardiovascular risk factors. Consequently, once cardiovascular risk becomes established and is driven by cumulative exposure to hypertension, dyslipidemia, and aging, circulating adipokine levels may lose their discriminatory relevance. In parallel, the growing literature on PreDMs highlights that cardiovascular risk begins early, before overt diabetes, but the translation of upstream metabolic mediators into downstream “risk-score strata” may be limited, especially when adipokines are measured at a single time point [57]. Thus, our findings support the interpretation that adipokines may act as upstream modulators of cardiometabolic health, whereas global risk scores represent downstream aggregates, reducing direct correspondence.

Importantly, the present findings do not negate the biological role of adipokines in cardiovascular physiology. Rather, they indicate that single-time-point adipokine measurements are insufficient to stratify individuals according to global cardiovascular risk, particularly when risk is estimated using multivariable prediction models. This interpretation is consistent with the concept that adipokines act as upstream modulators of cardiometabolic health, whereas cardiovascular risk scores represent downstream clinical aggregates.

Together, these results reinforce the notion that adipokines should be viewed as markers of early metabolic perturbation rather than surrogates of established cardiovascular risk, and they help explain why adipokine-based strategies have shown limited success in improving cardiovascular risk prediction beyond traditional models.

### 3.4. Discriminatory Performance of Adipokines: Biological Relevance Versus Clinical Utility

ROC analyses further highlight the distinction between biological relevance and clinical discrimination. In the overall cohort, adiponectin and omentin showed modest discriminatory performance for identifying metabolically obese phenotypes (AUC 0.704 and 0.710, respectively), whereas stratified analyses demonstrated poor discrimination in PreDM and limited feasibility in T2DM due to the predominance of the T2DM phenotype. This aligns with the modern consensus that metabolic phenotypes are not fixed entities and that binary phenotype classification can oversimplify continuous metabolic heterogeneity [51,52].

From a biomarker standpoint, these results are coherent with recent studies indicating that adipokines often contribute incremental information when integrated into multimarker or multivariable frameworks rather than functioning as standalone classifiers [45,49]. Therefore, our ROC findings should not be interpreted as “negative,” but rather as evidence that adipokines are better suited to mechanistic inference and risk biology than to dichotomous phenotype diagnostics.

### 3.5. Visceral Adiposity, Insulin Resistance, and Omentin in Early Dysglycemia

The incorporation of the VAI into multivariable models provides additional mechanistic insight into the determinants of circulating omentin levels in early dysglycemia. VAI has been proposed as a reliable surrogate marker of visceral adipose tissue dysfunction, integrating both anthropometric and lipid parameters to better reflect cardiometabolic risk compared with BMI alone [40,58]. More recent studies have confirmed its association with IR, subclinical inflammation, and incident diabetes [59].

In our PreDM cohort, VAI emerged as an independent predictor of circulating omentin concentrations, whereas BMI did not. This finding aligns with evidence suggesting that omentin is preferentially expressed in visceral adipose depots and may reflect qualitative alterations in adipose tissue function rather than total body adiposity [60]. Experimental and clinical data indicate that omentin levels are reduced in states of visceral adiposity and IR and are inversely associated with components of metabolic syndrome [23,61].

Interestingly, fasting insulin did not independently predict circulating omentin levels in adjusted models. This observation can suggest that composite indices integrating lipid and anthropometric measures (such as TyG or VAI) may better capture the cardiometabolic milieu associated with adipokine (omentin) dysregulation than isolated fasting insulin levels. In early dysglycemia, metabolic perturbations are multifactorial and may involve lipid toxicity, low-grade inflammation, and adipose tissue remodeling beyond hyperinsulinemia alone [62,63].

The higher VAI observed in women in our cohort may partly explain the independent contribution of sex in the multivariable models for omentin, reinforcing the concept that adipokine regulation is influenced not only by IR per se but also by sex-specific patterns of visceral adipose tissue dysfunction. Collectively, these findings underscore the importance of considering sex as a biological variable when evaluating adipokine–metabolic interactions in early dysglycemia. These findings support the concept that omentin is more closely linked to visceral adipose tissue dysfunction than to direct fasting insulin levels, reinforcing its potential role as a marker of early cardiometabolic risk in prediabetes [23,64,65].

### 3.6. Clinical Implications and Future Perspectives

The present findings have several important clinical and translational implications. First, the stage-dependent behavior of adipokines observed in this study suggests that adiponectin and omentin may be more informative in early dysglycemia than in established T2DM. Their stronger associations with IR-related indices in PreDMs indicate a potential role as early metabolic signals, reflecting subtle alterations in adipose tissue function before overt diabetes develops. This is consistent with recent literature arguing for early metabolic risk detection and intervention in PreDMs [57,66]. However, these observations should be interpreted as hypothesis-generating, as the cross-sectional design does not allow assessment of predictive performance or clinical decision thresholds.

Second, the lack of strong associations between adipokines and global cardiovascular risk scores, as well as their limited discriminatory performance for cardiometabolic phenotypes, highlights the restricted utility of single adipokine measurements in routine clinical risk stratification. These results reinforce the notion that adipokines should not be viewed as standalone diagnostic or prognostic markers, but rather as components of a broader biological context that includes insulin resistance, adiposity, inflammation, and lifestyle factors.

From a conceptual perspective, the present data align with emerging paradigms favoring continuous risk assessment rather than binary phenotype classification, particularly in individuals with PreDMs. Composite indices, such as TyG-based markers, low-cost tools that integrate metabolic and anthropometric information, may provide more actionable insights into early cardiometabolic risk assessment and into studies linking adipokines to metabolic burden [53,55]. In this framework, adipokines may serve as adjunctive biomarkers, offering mechanistic insight rather than direct clinical decision thresholds. Nonetheless, the present findings do not establish clinical utility, and further prospective validation is required before translation into routine practice.

Future work should prioritize longitudinal designs to assess whether baseline adipokines (alone or combined with TyG-derived indices) predict phenotype transitions and cardiometabolic outcomes [49,52]. Tracking changes in adiponectin and omentin over time may clarify whether early alterations predict progression from PreDMs to T2DM or the development of cardiovascular complications. In addition, integrating adipokines into multimarker panels, alongside inflammatory markers, genetic risk scores, and imaging-based assessments of adiposity, may enhance their translational value. Importantly, replication in larger, multi-center cohorts with longitudinal follow-up will be necessary to determine whether these associations translate into meaningful risk prediction.

Finally, the observed sex-specific influence on omentin levels underscores the need for sex-stratified analyses in future studies. Understanding how hormonal status, fat distribution, and sex-related differences in adipose tissue biology modulate adipokine signaling could refine personalized approaches to cardiometabolic risk assessment.

### 3.7. Strengths and Limitations

The present study has several notable strengths. First, it provides a comprehensive and integrative assessment of two key adipokines, adiponectin and omentin, across the spectrum of dysglycemia, combining metabolic phenotyping, IR surrogates (including TyG-derived indices), cardiovascular risk stratification, and ROC-based discrimination analyses. This multidimensional approach allowed for a nuanced evaluation of adipokine behavior beyond simple group comparisons. Second, the inclusion of both PreDM and newly diagnosed T2DM enabled the identification of stage-dependent differences in adipokine–metabolic relationships, highlighting early metabolic alterations that may be obscured in established disease. The use of composite metabolic indices, such as TyG-based markers, further strengthened the metabolic relevance of the analyses. This framework aligns with recent calls to evaluate metabolic risk using multidimensional models rather than single biomarkers [51,52,67].

Several limitations should also be acknowledged. The cross-sectional design precludes causal inferences and limits the ability to assess temporal changes in adipokine levels or their predictive value for disease progression. Residual confounding cannot be excluded, as lifestyle factors (dietary patterns, physical activity, smoking status), medication use, and menopausal status were not comprehensively controlled for. In addition, single-time-point adipokine measurement and the lack of inflammatory profiling limit mechanistic depth, which is important because recent evidence highlights the coupling between TyG, inflammation, and adipokine imbalance [55]. In addition, visceral adiposity was estimated using surrogate indices (e.g., VAI) rather than imaging-based techniques (e.g., MRI or CT), which may introduce measurement imprecision.

The sample size, particularly in stratified and phenotype-based analyses, may have limited statistical power to detect modest associations, especially in the T2DM group, where metabolic phenotypic variability was reduced. Given the number of correlation and regression analyses performed, the possibility of type I error cannot be entirely excluded. Furthermore, the lack of concurrent assessment of inflammatory markers and adipokine receptor expression restricts mechanistic interpretation.

Despite these limitations, the present findings provide biologically plausible and internally consistent evidence that adipokines may be more closely linked to early metabolic dysfunction than to established cardiometabolic phenotypes or global cardiovascular risk. Nevertheless, their clinical applicability requires confirmation in prospective and mechanistic studies.

## 4. Materials and Methods

### 4.1. Patient Selection

This retrospective cohort study involved 128 consecutive patients newly diagnosed with T2DM at a single university hospital in Craiova, Dolj, Romania. Additionally, 100 PreDM patients who met the inclusion criteria, based on factors such as age, gender distribution, and urban/rural residence, served as the control group. The study adhered to the Declaration of Helsinki and received approval from the Ethics Committee of the Clinical Municipal Hospital Filantropia Craiova (no. 887/15 January 2024).

We chose individuals aged 18 or older with newly diagnosed T2DM from the Diabetes, Nutrition, and Metabolic Diseases Departments at the Clinical Municipal Hospital Filantropia Craiova. All participants provided informed consent and voluntarily participated.

We excluded from the study group patients with T2DM diagnosed with microvascular complications, including peripheral polyneuropathy (*n* = 28), kidney disease (*n* = 14) [68], and retinopathy (*n* = 16). It excluded individuals under 18, pregnant women, people with type 1 diabetes, recent infections or inflammatory diseases, current infections, inflammatory conditions, or cancer.

Participants were divided into two groups based on cutoff values for FPG, plasma glucose after 2 h, and HbA1c, according to the latest American Diabetes Association guidelines [69]. We applied these cutoff points consistently, regardless of whether glucose levels were measured in plasma or serum. The first state was T2DM, indicated by clinical markers (HbA1c level above 6.5%, an FPG level of 7.0 mmol/L or higher, a random blood glucose level of 11.1 mmol/L or higher, or a two-hour blood glucose level exceeding 11.1 mmol/L after an OGTT), self-report, use of glucose-lowering medications, or previous diagnosis. The second state was PreDM, indicated clinically (an HbA1c level from 5.7% to just below 6.5%; an FPG level between 5.6 mmol/L and 7.0 mmol/L; or an OGTT result from 7.8 mmol/L to 11.0 mmol/L), by not meeting the other criteria for T2DM, or by a healthcare professional.

Data on physical measurements, medical conditions, lab results, lifestyle factors, demographic variables (age, gender, income, education-into lower (up to and including year 12) or higher (more than year 12)), and health behaviors (smoking—current smoker, former smoker, or non-smoker; alcohol; family health history; physical activity/inactive (<150 min/week) or moderately active to active (≥150 min/week)) were collected via an interview questionnaire.

### 4.2. Evaluation of Different Obesity-Related Indices (BMI, WHR, WHtR)

The BMI was calculated from participants’ weight and height. The formula for BMI is BMI = weight (kilograms)/height^2^ (meters). The patient’s nutritional status was assessed using BMI, according to the WHO criteria [70]. BMI was categorized into normal weight (18.5–22.9 kg/m^2^), overweight (23.0–25.0 kg/m^2^), and obese (>25.0 kg/m^2^). A weight scale was used to measure weight, while height was measured with a measuring stick attached to the weight scale.

Measurements of the waist circumference (WC) were taken at the midpoint between the lower border of the rib cage and the upper iliac crest, while the hip circumference (HC) was measured over the femoral trochanters. Abdominal obesity was assessed using the waist-to-hip ratio (WHR), calculated as WC (cm)/HC (cm). The waist-to-height ratio (WHtR) was also used to evaluate visceral adiposity, calculated as WC (cm)/height (m).

### 4.3. Metabolic Syndrome Definition and Cardiometabolic Phenotypes

Metabolic syndrome (MetS) was defined based on the 2009 Harmonized criteria, which require at least three out of five specific diagnostic criteria/risk factors [71]:

- Waist circumference: >102 cm (male); >88 cm (female);
- Serum FPG: ≥100 mg/dL or use of hypoglycemic drugs;
- Serum TG: ≥150 mg/dL or use of triglyceride-lowering drugs;
- Reduced HDL-C values: ≤40 mg/dL (males) or ≤50 mg/dL (females), or use of HDL-C-raising drugs;
- Elevated blood pressure: SBP ≥ 130 mmHg or DBP ≥ 85 mmHg, or use of blood pressure-lowering drugs.

In evaluating patients, we have used a study by Held et al., which found that patients with stable coronary heart disease exhibited a progressive elevation in cardiometabolic and inflammatory risk markers as their BMI exceeded 25 kg/m^2^ [72]. Using a BMI of <25 kg/m^2^ as normal weight and ≥25 kg/m^2^ as overweight or obese, we divided the patients into 4 cardiometabolic phenotypes based on their BMI and the presence of MetS, as adapted from Soheilifard et al. [4]:

- MHNW—BMI < 25 kg/m^2^ and <3 MetS criteria;
- MUHNW—BMI < 25 kg/m^2^ and ≥3 MetS criteria;
- MHO—BMI ≥ 25 kg/m^2^ and <3 MetS criteria;
- MUHO—BMI ≥ 25 kg/m^2^ and ≥3 MetS criteria.

Using these criteria, we then defined the following cohorts according to metabolic phenotypes:

- PreDM cohort: the MHNW (*n* = 28), MUHNW (*n* = 32), and MUHO (*n* = 40) phenotypes;
- T2DM cohort: the MHO (*n* = 72) and MUHO (*n* = 56) phenotypes.

### 4.4. Evaluation of Various Indices of Insulin Resistance and Lipid-Related Risk Factors (HOMA-IR, QUICKI, TG/HDL-C, AIP, and LAP)

To evaluate insulin resistance and the metabolic profile, we used the formulas:

- HOMA-IR: (Insulin [µU/mL] × Glucose [mg/dL])/405 [73];
- QUICKI: 1/((log (Insulin [µU/mL]) + log (Glucose [mg/dL])) [74];
- AIP: log10 (TG/HDL-C) [75,76];
- Remnant Cholesterol (RC): Total Cholesterol—LDL-C—HDL-C;
- Non-HDL-C: Total Cholesterol − HDL-C;
- TyG index: ln([TG (mg/dL) × Glucose (mg/dL)]/2) [77,78,79];
- TyG-BMI: TyG × BMI [77];
- TyG-WHtR: TyG × WHtR [78];
- TyG-WC: TyG × WC [79].

### 4.5. Cardiovascular Risk Assessment

Cardiovascular risk was estimated using the 10-year general CVD risk from the FRS and the WHO CVD risk scores:

- FRS for general CVD was calculated using the model proposed by D’Agostino et al., which estimates the 10-year probability of a significant cardiovascular event. The algorithm incorporates age, sex, SBP, treatment for hypertension, TC, HDL-C, smoking status, and diabetes status; sex-specific coefficients were applied, and results were expressed as percentage risk [80].
  - L_Men_ = β × ln(Age) + β × ln(TC) + β × ln(HDL-C) + β × ln(SBP) + β × Treated for blood pressure + β × Smoker + β × ln(Age) × ln(TC) + β × ln(Age) × Smoker + β × ln(Age) × ln(Age)—172.300168 [81];
  - L_Women_ = β × ln(Age) + β × ln(TC) + β × ln(HDL-C) + β × ln(SBP) + β × Treated for blood pressure + β × Smoker + β × ln(Age) × ln(TC) + β × ln(Age) × Smoker—146.5933061 [81];
- The WHO CVD risk prediction charts (non-laboratory model) for the Eastern Europe region estimate 10-year cardiovascular risk based on age, sex, SBP, smoking status, and diabetes status. Participants are classified into five risk categories: <5%, 5–9%, 10–19%, 20–29%, and ≥30%. For statistical analyses requiring continuous variables, categorical WHO risk estimates are converted into approximate percentages using the midpoint of each category, a common method in epidemiological research [82,83].

### 4.6. Laboratory Investigations

After acquiring the anthropometric data, we conducted more thorough evaluations of the subjects in the lab.

#### Sample Collection—Each Patient Provided Two Blood Samples Collected in Separate Tubes During the Biological Sampling Process

- Two 5 mL tubes with patient blood (Becton Dickinson Vacutainer, Franklin Lakes, NJ, USA) were centrifuged at 3000× *g* for 10 min in a Hermle centrifuge (Hermle AG, Gosheim, Baden-Württemberg, Germany) within 4 h of clotting. Serum from one tube was aliquoted into sealed, labeled vials and stored at −20 °C to −80 °C. Care was taken to avoid freeze–thaw cycles in order to maintain the integrity of the samples. Frozen serum samples were thawed to room temperature before analysis. Immunological assessments were performed using these aliquots, while biochemical tests were performed on serum from the second tube.

Biochemical Investigations—Biochemical Parameters Were Measured Using the ARCHITECT c4000 Analyzer (Abbott Laboratories, Abbott Park, IL, USA) with Photometric, Enzymatic, and Potentiometric Methods, Depending on the Analyte. Serum or Plasma Analysis Was Performed According to the Manufacturer’s Procedures. Measurements Complied with Calibration and Quality Control Protocols.

### 4.7. Immunological Assessment

Serum biomarker levels were measured using an ELISA kit from Elabscience (Houston, TX, USA) based on the sandwich ELISA principle, at the Laboratory of Immunology from the University of Medicine and Pharmacy of Craiova.

- Human INS(Insulin) ELISA Kit (Cat. No.: E-EL-H2665; Sensitivity: 0.47 μIU/mL; Detection Range: 0.78–50 μIU/mL; Specificity: No significant cross-reactivity or interference between Human INS and analogues was observed; Intra-/Inter-Assay CV (%): Coefficient of variation is <10%; https://www.elabscience.com/p/human-ins-insulin-elisa-kit--e-el-h2665 (accessed on 5 December 2025);
- Human ITLN1(Intelectin 1/Omentin) ELISA Kit (Cat. No.: E-EL-H2028; Sensitivity: 0.38 ng/mL; Detection Range: 0.63–40 ng/mL; Specificity: No significant cross-reactivity or interference between Human ITLN1 and analogues was observed; Intra-/Inter-Assay CV (%): Coefficient of variation is <10%; https://www.elabscience.com/p/human-itln1-intelectin-1-omentin-elisa-kit--e-el-h2028 (accessed on 5 December 2025);
- Human ADP/Acrp30(Adiponectin) ELISA Kit (Cat. No.: E-EL-H6122; Sensitivity: 0.1 ng/mL; Detection Range: 0.16–10 ng/mL; Specificity: No significant cross-reactivity or interference between Human ADP and analogues was observed; Intra-/Inter-Assay CV (%): Coefficient of variation is <10%; https://www.elabscience.com/p/human-adp-acrp30-adiponectin-elisa-kit--e-el-h6122 (accessed on 5 December 2025).

For optical density detection, we used a standard spectrophotometer, the Asys Expert Plus Microplate Reader (ASYS Hitech GmbH, Eugendorf, Austria), to measure at 450 nm, following the manufacturer’s instructions.

### 4.8. Statistical Analysis

Data were analyzed using GraphPad Prism 10.6.1 (892) (GraphPad Software, San Diego, CA, USA). Continuous variables were tested for normality using the Shapiro–Wilk test and presented as mean ± SD or median [IQR], as appropriate.

To evaluate the relationships between circulating adipokines and metabolic, atherogenic, and cardiovascular risk indices, correlation analyses were performed separately in participants with PreDM and those with newly diagnosed T2DM. Given the non-normal distribution of most biochemical variables, as assessed by the Shapiro–Wilk test, Spearman’s rank correlation coefficients (*ρ*) were calculated. Circulating adiponectin and omentin levels were correlated with lipid-derived and IR-related indices, including the TG/HDL-C, AIP, TyG, and TyG-based composite indices incorporating anthropometric measures (TyG-BMI, TyG-WC, and TyG-WHtR). In addition, to address visceral adiposity and global cardiovascular risk, correlations were extended to include the VAI and validated cardiovascular risk scores, namely the Framingham Risk Score (10-year risk; FRS 10y) and the WHO CVD risk score.

Correlation analyses were conducted independently within each glycemic group (PreDM and T2DM), and two-tailed *p*-values < 0.05 were considered statistically significant. To evaluate whether the associations between circulating adipokines and insulin resistance were independent of demographic and adiposity-related factors, multivariable linear regression (MLR) analyses were conducted in the PreDM group, where significant univariate correlations were identified. Separate regression models were constructed using omentin or adiponectin as the dependent variable. The TyG index was included as the primary independent variable in the main models. Covariates were selected a priori based on biological plausibility and included age, sex, and measures of adiposity. For omentin, Model A evaluated the association between TyG and circulating omentin levels adjusted for age, sex, and BMI. To specifically assess visceral adiposity, Model B replaced BMI with the VAI. In an additional exploratory model (Model C), fasting insulin was entered as the primary metabolic predictor, adjusted for age, sex, and VAI, to assess whether direct measures of insulin resistance independently predicted omentin levels. All predictors were entered simultaneously using the enter method. Unstandardized regression coefficients (β), 95% confidence intervals (CI), model sample size (n), and coefficient of determination (R^2^) were reported. Statistical significance was defined as *p* < 0.05.

To explore the relationship between circulating adipokines and global cardiovascular risk, adiponectin and omentin levels were evaluated across cardiovascular risk categories derived from validated risk prediction models. Participants were stratified according to their 10-year cardiovascular risk estimated by the FRS into three categories: low risk (<10%), intermediate risk (10–19%), and high risk (≥20%). Circulating adipokine levels were compared descriptively across these categories and are presented as median [IQR].

To evaluate the ability of circulating adipokines to discriminate between cardiometabolic phenotypes, ROC curve analyses were performed. Separate analyses were conducted for adiponectin and omentin. Metabolic phenotypes were defined according to established criteria based on BMI and metabolic health status, with a primary focus on distinguishing metabolically obese phenotypes from non-metabolically obese phenotypes. ROC analyses were performed in the overall study population and, where appropriate, stratified by glycemic status (PreDM and newly diagnosed T2DM). The discriminatory performance of each adipokine was quantified as the area under the ROC curve (AUC), with corresponding 95% CI. An AUC value of 0.5 indicated no discriminatory ability, whereas values approaching 1.0 reflected increasing discrimination. Optimal cut-off values were explored using the Youden index, defined as the maximum value of sensitivity + specificity − 1. Sensitivity, specificity, and AUC values were reported descriptively to assess the potential clinical utility of adiponectin and omentin as biomarkers for discriminating metabolically obese phenotypes.

## 5. Conclusions

In conclusion, the present study suggests that circulating adipokines exhibit stage-dependent associations with metabolic dysfunction across the spectrum of dysglycemia. Both adiponectin and omentin were lower in newly diagnosed T2DM compared with PreDM, supporting a progressive alteration of adipokine profiles during worsening metabolic status. In individuals with PreDM, omentin showed an inverse association with IR, as reflected by the TyG index; however, this relationship was attenuated after multivariable adjustment, indicating the influence of demographic and adiposity-related factors, such as sex and visceral adiposity. In contrast, adipokine–metabolic associations were largely absent in newly diagnosed T2DM. Circulating adipokines also showed limited variation across cardiometabolic phenotypes and cardiovascular risk categories and demonstrated modest to poor discriminatory performance for metabolically obese phenotypes. Given the cross-sectional design and subgroup stratification, these findings should be interpreted cautiously and considered exploratory. Overall, the results suggest that adipokines may reflect early adipose tissue-related metabolic perturbations rather than serve as standalone clinical biomarkers. Larger longitudinal studies integrating multimarker and mechanistic approaches are required to clarify their predictive value and clinical relevance.

## Figures and Tables

**Table 1:** Metabolic, Cardiovascular, and Adipokine Characteristics of Participants with PreDM and Newly Diagnosed T2DM.

Variable	PreDM (*n* = 100)	T2DM (*n* = 128)	*p* Value
Demographic variables			
Age (years) (mean ± SD)	55.73 ± 9.74	55.41 ± 11.53	0.185
Areas (Rural/Urban)	37/63	55/73	0.362
Sex (Male/Female)	52/48	65/63	0.862
Education Yes/No (n)	61/39	89/39	0.177
Health and lifestyle-related factors			
Drinking Yes/No (n)	52/48	42/86	0.003
Smoking Yes/No (n)	57/43	46/82	0.002
Physical activity Yes/No (n)	56/44	62/66	0.256
Hepatosteatosis n (%)	68 (68.00%)	79 (61.72%)	0.325
Dyslipidemia n (%)	64 (64.00%)	81 (63.28%)	0.920
Hypertension n (%)	67 (67.00%)	98 (76.56%)	0.109
SBP (mmHg) (mean ± SD)	127.70 ± 11.33	153.00 ± 11.71	<0.0001
Anthropometric characteristics			
BMI (kg/m^2^) (median [IQR])	26.65 [24.77–29.30]	32.74 [30.03–37.97]	<0.0001
Weight (kg) (median [IQR])	81.00 [71.00–90.75]	88.50 [74.00–100.00]	0.002
Height (cm) (mean ± SD)	168.40 ± 10.50	164.10 ± 9.65	0.006
Waist circumference (cm) (median [IQR])	105.60 [96.00–113.60]	111.00 [104.00–118.00]	0.120
Hip circumference (cm) (mean ± SD)	106.60 ± 14.70	111.20 ± 11.96	0.012
WHR (mean ± SD)	0.98 ± 0.15	0.92 ± 0.14	0.003
WHtR (mean ± SD)	0.62 ± 0.08	0.62 ± 0.08	0.747
Glycemic and lipid profile			
HbA1c (%) (mean ± SD)	5.95 ± 0.40	9.05 ± 1.87	<0.0001
FPG (mg/dL) (median [IQR])	98.00 [85.00–107.80]	164.80 [138.00–188.30]	<0.0001
TG (mg/dL) (median [IQR])	152.50 [116.80–194.00]	176.30 [119.00–198.50]	0.828
TC (mg/dL) (mean ± SD)	213.00 ± 49.49	194.90 ± 55.56	0.010
HDL-C (mg/dL) (mean ± SD)	49.34 ± 13.40	49.36 ± 13.22	0.991
LDL-C (mg/dL) (mean ± SD)	140.80 ± 35.43	129.00 ± 41.53	0.022
Non-HDL-C (mg/dL) (mean ± SD)	163.70 ± 47.91	145.60 ± 55.17	0.009
RC (TC—HDL-C—LDL-C) (mg/dL) (mean ± SD)	22.83 ± 41.51	16.52 ± 52.82	0.313
Metabolic Parameters			
Insulin (µU/mL) (median [IQR])	5.34 [3.21–10.28]	48.22 [42.39–50.71]	<0.0001
HOMA-IR (median [IQR])	1.30 [0.83–2.28]	18.44 [15.83–21.25]	<0.0001
QUICKI (median [IQR])	0.16 [0.14–0.17]	0.11 [0.11–0.12]	<0.0001
TG/HDL-C (median [IQR])	3.23 [2.29–4.28]	3.31 [2.54–4.18]	0.806
AIP (log10 TG/HDL) (mean ± SD)	0.51 ± 0.23	0.52 ± 0.24	0.829
VAI (median [IQR])	3.38 [2.29–4.77]	2.18 [1.49–3.16]	<0.0001
TyG (mean ± SD)	9.07 ± 0.58	9.23 ± 0.57	0.041
TyG-BMI (mean ± SD)	244.70 ± 38.35	319.90 ± 57.38	<0.0001
TyG-WC (mean ± SD)	942.60 ± 147.10	939.20 ± 141.20	0.862
TyG-WHtR (mean ± SD)	5.60 ± 0.83	5.73 ± 0.84	0.246
Cardiovascular Risk Assessment			
FRS 10y General CVD (%) (median [IQR])	12.69 [7.69–19.90]	26.20 [16.83–44.17]	<0.0001
WHO CVD risk (%) (median [IQR])	12.00 [10.00–16.75]	20.00 [15.00–22.00]	<0.0001
Cardiometabolic Phenotypes, *n* (%)			
MHNW	28 (28.00%)	-	-
MUHNW	32 (32.00%)	-	-
MHO	-	72 (56.25%)	-
MUHO	40 (40.00%)	56 (43.75%)	0.023
Adipokine concentrations			
Adiponectin (ng/dL) (median [IQR])	30.30 [25.19–34.39]	22.05 [16.75–26.00]	<0.0001
Omentin (ng/mL) (median [IQR])	38.84 [33.94–41.30]	25.72 [19.20–28.47]	<0.0001

SBP: systolic blood pressure; BMI: body mass index; HbA1c: glycosylated hemoglobin A1c; FPG: fasting plasma glucose; TC: total cholesterol; TG: total triglycerides; HDL-C: high-density lipoprotein cholesterol; LDL-C: low-density lipoprotein cholesterol; HOMA-IR: Homeostatic Model Assessment of Insulin Resistance; QUICKI: Quantitative Insulin Sensitivity Check Index; AIP: atherogenic index of plasma; VAI: Visceral Adiposity Index; WC: waist circumference; MHNW: Metabolically Healthy Normal Weight; MUHNW: Metabolically Unhealthy Normal Weight; MHO: Metabolically Healthy Obese; MUHO: Metabolically Unhealthy Obese; FRS: Framingham Risk Score; WHO CVD: World Health Organization cardiovascular disease risk.

**Table 2:** Gender-based differences in adipokines, metabolic parameters, and cardiovascular risk in PreDM and T2DM.

Parameters		PreDM		T2DM		
	Male (*n* = 52)	Female (*n* = 48)	*p* Value	Male (*n* = 65)	Female (*n* = 63)	*p* Value
Adiponectin (ng/dL) (median [IQR])	31.84 [24.75–38.72]	28.89 [26.95–30.96]	0.019	21.90 [16.98–26.03]	22.20 [16.05–25.85]	0.238
Omentin (ng/mL) (median [IQR])	41.42 [38.54–43.50]	36.29 [30.36–41.74]	0.013	25.79 [16.09–33.60]	25.47 [19.23–26.84]	0.786
Insulin (µU/mL) (median [IQR])	4.75 [2.98–8.90]	8.67 [3.66–12.70]	0.0525	49.62 [42.81–51.56]	49.96 [43.68–52.17]	0.684
HOMA-IR (median [IQR])	1.51 [1.09–2.00]	1.83 [1.00–2.99]	0.158	14.46 [10.81–18.71]	14.08 [12.27–17.93]	0.861
QUICKI (median [IQR])	0.163 [0.150–0.176]	0.153 [0.141–0.171]	0.026	0.11 [0.11–0.12]	0.11 [0.11–0.12]	0.913
FRS 10y General CVD (%) (median [IQR])	16.63 [9.53–26.38]	9.24 [6.15–13.83]	<0.0001	34.68 [22.94–50.09]	21.91 [12.08–31.59]	<0.0001
WHO CVD risk (%) (median [IQR])	12.50 [8.25–17.00]	10.00 [9.40–14.75]	0.016	20.00 [17.00–22.00]	18.00 [15.00–22.00]	0.018
TG/HDL-C (median [IQR])	3.43 [2.11–4.79]	3.19 [2.58–4.00]	0.679	3.20 [2.37–4.17]	3.38 [2.54–4.44]	0.479
AIP (log10 TG/HDL) (mean ± SD)	0.51 ± 0.22	0.51 ± 0.24	0.971	0.51 ± 0.24	0.53 ± 0.23	0.596
VAI (median [IQR])	3.09 [2.13–4.26]	3.87 [2.94–5.32]	0.007	1.79 [1.25–2.45]	2.78 [2.13–3.60]	<0.0001
TyG (mean ± SD)	8.85 ± 0.40	8.94 ± 0.51	0.338	9.40 ± 0.46	9.51 ± 0.49	0.182
TyG-BMI (mean ± SD)	232.62 ± 40.53	248.60 ± 35.93	0.039	323.20 ± 62.09	332.30 ± 53.48	0.379
TyG-WC (mean ± SD)	899.62 ± 133.91	949.93 ± 134.14	0.064	949.50 ± 137.00	971.90 ± 120.60	0.326
TyG-WHtR (mean ± SD)	5.37 ± 0.72	5.92 ± 0.82	0.046	5.69 ± 0.74	6.05 ± 0.80	0.009

**Table 3:** Comparing the Adipokines and Metabolic Parameters Levels According to the cardiometabolic phenotypes in the PreDM Group.

Parameter	MHNW (*n* = 28)	MUHNW (*n* = 32)	MUHO (*n* = 40)	Factor	F (DFn, DFd)	*p* Value
Adiponectin (ng/dL) median [IQR]	29.34	27.81	29.74	Row factor	F(71,55) = 0.955	0.576
	24.18–30.93	24.84–31.36	27.83–31.48	Column factor	F(1,55) = 0.183	0.671
Omentin (ng/mL) median [IQR]	38.73	37.85	38.57	Row factor	F(71,55) = 0.741	0.883
	38.61–39.91	29.20–40.22	32.16–41.74	Column factor	F(1,55) = 0.044	0.835
Insulin (µU/mL) median [IQR]	4.52	4.39	10.15	Row factor	F(71,55) = 0.863	0.723
	2.77–6.15	3.22–9.05	3.94–15.38	Column factor	F(1,55) = 3.500	0.046
HOMA-IR median [IQR]	1.22	1.50	2.34	Row factor	F(71,55) = 1.342	0.128
	0.86–1.61	1.01–2.05	1.53–3.92	Column factor	F(1,55) = 2.171	0.146
QUICKI median [IQR]	0.168	0.161	0.148	Row factor	F(71,55) = 0.989	0.522
	0.159–0.211	0.150–0.173	0.136–0.167	Column factor	F(2,31) = 4.833	0.032

**Table 4:** Comparing the Adipokines and Metabolic Parameters Levels According to the cardiometabolic phenotypes in the T2DM Group.

Parameter	MHO (n = 72)	MUHO (n = 56)	Factor	F (DFn, DFd)	*p* Value
Adiponectin (ng/dL) median [IQR]	22.15	22.05	Row factor	F(71,55) = 0.955	0.576
	16.62–26.11	16.77–25.96	Column factor	F(1,55) = 0.183	0.671
Omentin (ng/mL) median [IQR]	25.72	25.03	Row factor	F(71,55) = 0.741	0.883
	19.24–26.81	16.22–38.65	Column factor	F(1,55) = 0.044	0.835
Insulin (µU/mL) median [IQR]	49.73	49.88	Row factor	F(71,55) = 0.869	0.723
	44.92–51.71	36.33–51.73	Column factor	F(1,55) = 3.500	0.046
HOMA-IR median [IQR]	14.17	14.28	Row factor	F(71,55) = 1.342	0.128
	11.99–18.41	11.01–18.00	Column factor	F(1,55) = 2.171	0.146
QUICKI median [IQR]	0.112	0.113	Row factor	F(71,55) = 0.988	0.522
	0.110–0.113	0.110–0.117	Column factor	F (1,55) = 4.833	0.032

**Table 5:** Spearman correlations between circulating adipokines and metabolic and atherogenic indices in participants with prediabetes (PreDM).

Adipokine	Index	Spearman Coefficient (*ρ*)	*p*-Value
Adiponectin	TG/HDL-C	−0.077	0.448
Adiponectin	AIP	−0.077	0.448
Adiponectin	VAI	0.029	**0.771**
Adiponectin	TyG index	−0.162	**0.107**
Adiponectin	TyG-BMI	0.052	0.607
Adiponectin	TyG-WC	−0.051	0.617
Adiponectin	TyG-WHtR	−0.089	0.377
Adiponectin	FRS 10y	0.203	0.042
Adiponectin	WHO CVD	−0.032	0.750
Omentin	TG/HDL-C	−0.084	0.405
Omentin	AIP	−0.084	0.405
Omentin	VAI	−0.108	**0.285**
Omentin	TyG index	−0.197	**0.050**
Omentin	TyG-BMI	0.149	0.140
Omentin	TyG-WC	−0.090	0.370
Omentin	TyG-WHtR	−0.069	0.496
Omentin	FRS 10y	0.189	0.060
Omentin	WHO CVD	0.152	0.132

Spearman’s rank correlation coefficients (*ρ*) were used due to the non-normal distribution of the analyzed variables. Significant correlations (*p* < 0.05) are highlighted in bold. AIP: atherogenic index of plasma; TyG: triglyceride–glucose index; VAI: visceral adiposity index; FRS 10y: Framingham Risk Score (10-year risk); WHO CVD: World Health Organization cardiovascular disease risk score; WC: waist circumference; WHtR: waist-to-height ratio.

**Table 6:** Spearman correlations between circulating adipokines and metabolic and atherogenic indices in participants with type 2 diabetes (T2DM).

Adipokine	Index	Spearman Coefficient (*ρ*)	*p*-Value
Adiponectin	TG/HDL-C	−0.080	0.368
Adiponectin	AIP	−0.080	0.368
Adiponectin	VAI	−0.070	0.452
Adiponectin	TyG index	0.021	0.815
Adiponectin	TyG-BMI	−0.139	0.117
Adiponectin	TyG-WC	−0.092	0.303
Adiponectin	TyG-WHtR	−0.136	0.127
Adiponectin	FRS 10y	0.040	0.657
Adiponectin	WHO CVD	−0.050	0.085
Omentin	TG/HDL-C	−0.003	0.975
Omentin	AIP	−0.003	0.975
Omentin	VAI	−0.030	0.747
Omentin	TyG index	−0.078	0.381
Omentin	TyG-BMI	−0.018	0.840
Omentin	TyG-WC	−0.031	0.730
Omentin	TyG-WHtR	−0.029	0.747
Omentin	FRS 10y	−0.150	0.565
Omentin	WHO CVD	0.090	0.329

Spearman’s rank correlation coefficients (*ρ*) were used due to non-normal distribution of the analyzed variables.

**Table 7:** Multivariable linear regression analyses of circulating adipokines in the PreDM group.

Model	Dependent	Variable	β	95% CI	*p*-Value	R^2^
Model A	Omentin	const	39.15	1.87 to 76.43	0.039	0.105
		TyG	−2.48	−6.38 to 1.41	0.208	
		Age (y)	0.04	−0.14 to 0.22	0.628	
		Sex (M/F)	2.12	−1.39 to 5.63	0.233	
		BMI (kg/m^2^)	0.58	0.17 to 0.99	0.006	
Model B	Omentin	const	94.24	54.32 to 134.17	<0.0001	0.122
		TyG	−7.64	−12.44 to −2.84	0.002	
		Age (y)	0.09	−0.09 to 0.27	0.325	
		Sex (M/F)	1.76	−1.67 to 5.19	0.311	
		VAI	0.93	0.34 to 1.51	0.002	
Model C	Omentin	const	30.55	19.79 to 41.31	<0.0001	0.086
		Insulin (µU/mL)	0.35	0.06 to 0.64	0.017	
		Age (y)	0.03	−0.15 to 0.21	0.731	
		Sex (M/F)	2.48	−1.06 to 6.02	0.168	
		VAI	−0.01	−0.58 to 0.56	0.963	

**Table 8:** ROC analysis of circulating adipokines for identifying metabolically obese phenotype.

Cohort	Biomarker	AUC	95% CI	Cut-Off	Sensitivity (%)	Specificity (%)
Overall	Adiponectin	0.704	0.628–0.774	26.37 µg/mL	70	67
Overall	Omentin	0.710	0.636–0.781	28.76 ng/mL	61	88
PreDM	Adiponectin	0.431	0.323–0.544	29.87 µg/mL	63	45
PreDM	Omentin	0.461	0.345–0.573	38.55 ng/mL	50	58

## Data Availability

The data used to support the findings of this study are available from the corresponding author upon reasonable request.
